# Experimental Analysis of the Influence of Carrier Layer Material on the Performance of the Control System of a Cantilever-Type Piezoelectric Actuator

**DOI:** 10.3390/ma17010096

**Published:** 2023-12-24

**Authors:** Dariusz Grzybek

**Affiliations:** Faculty of Mechanical Engineering and Robotics, AGH University of Krakow, al. A. Mickiewicza 30, 30-059 Krakow, Poland; dariusz.grzybek@agh.edu.pl; Tel.: +48-126173080

**Keywords:** piezoelectric actuator, macro fiber composite, sandwich beam, LQR control algorithm

## Abstract

The subject of this article is an experimental analysis of the control system of a composite-based piezoelectric actuator and an aluminum-based piezoelectric actuator. Analysis was performed for both the unimorph and bimorph structures. To carry out laboratory research, two piezoelectric actuators with a cantilever sandwich beam structure were manufactured. In the first beam, the carrier layer was made of glass-reinforced epoxy composite (FR4), and in the second beam, it was made of 1050 aluminum. A linear mathematical model of both actuators was also developed. A modification of the method of selecting weights in the LQR control algorithm for a cantilever-type piezoelectric actuator was proposed. The weights in the R matrix for the actuator containing a carrier layer made of stiffer material should be smaller than those for the actuator containing a carrier layer made of less stiff material. Additionally, regardless of the carrier layer material, in the case of a bimorph, the weight in the R matrix that corresponds to the control voltage of the compressing MFC patch should be smaller than the weight corresponding to the control voltage of the stretching MFC patch.

## 1. Introduction

A piezoelectric actuator is a device that uses the inverse piezoelectric effect to convert electrical energy into mechanical energy: because of this energy conversion, motion of the mechanical component of the actuator is generated [1]. One of the mechanical components used in piezoelectric actuators is the cantilever beam [2]. Two basic types of cantilever beam structure can be distinguished: unimorph and bimorph. The unimorph is a structure in which there is one layer of piezoelectric material and one carrier layer. The bimorph is a structure with two layers of piezoelectric material and one carrier layer [3], or with two layers of piezoelectric material alone [4]. Some researchers use the name “triple-layer” instead of the name “bimorph” [5]. In the unimorph and bimorph structures of the cantilever beam, the layers are usually glued together [6]. In the case of a structure containing a carrier layer, the motion of the cantilever beam is generated by creating tensile or compressive stresses in this carrier layer through the interaction of the piezoelectric layer (unimorph) or two layers (bimorph).

In both the unimorph and the bimorph, the piezoelectric layers can be made of different materials. The piezoelectric materials used can be divided into three main groups: (1) piezoelectric ceramics, usually lead zirconate titanate (PZT) [7]; (2) piezoelectric composites, usually type P1 macro fiber composite (MFC) made from PZT fibers and warp of nonpiezoelectric polymers [8]; and (3) piezoelectric polymers, usually polyvinylidene fluoride (PVDF) [9]. The first fundamental difference in the use of these piezoelectric materials is due to the relationship between energy conversion efficiency and brittleness. Piezoelectric ceramics are characterized by the highest energy conversion efficiency but are at the same time the most fragile compared to composites or polymers [10]. On the other hand, piezoelectric polymers are the most flexible but have the lowest energy conversion efficiency compared to ceramics and composites [9]. Composites have lower energy conversion efficiency than ceramics but are more resistant to destruction due to deformations [11]. The second fundamental difference in the use of these piezoelectric materials in the cantilever beams of the actuators results from the relationship between the direction of the stress generated in the carrier layer by the piezoelectric layer or layers and the direction of polarization of the piezoelectric layer or layers. When piezoelectric ceramics and polymers are used, the polarization direction of the piezoelectric layer or layers is perpendicular to the direction of stress generated in the carrier layer. When the composite MFC type P1 is used, the direction of stress is parallel to the direction of polarization. This difference leads to the fact that the conversion of electrical energy into mechanical energy in actuators with the use of PZT is described by the piezoelectric coefficient d_31_, and in actuators with the use of MFC type P1 by d_33_. Nguyen et al. [12] also noticed that MFC has better dynamic actuation than the bulk PZT type for the range of high frequency.

The carrier layer of the cantilever beam in piezoelectric actuators is made of materials that can be divided into two main groups: (1) metals, and (2) composites. The metals used in the cantilever structure are primarily aluminum alloys [13], brass [14], beryllium [15], and steel [16]. The composite used is primarily glass-reinforced epoxy composite (FR4) [17]. The use of a stiffer material in the carrier layer leads to a decrease in the value of the cantilever beam tip motion generated [18]. The tip motion of the cantilever beam made from aluminum is larger in comparison to the motion of actuators made from steel or copper; however, this difference decreases as the thickness of the carrier layer decreases [19]. In general, for the same geometrical dimension and under the same applied electric field, the lower the stiffness of the material of the carrier layer in the cantilever beam of the piezoelectric actuator, the greater displacements of this cantilever beam tip are generated. On the other hand, the application of a carrier layer with greater stiffness leads to a generation of larger blocking forces [20]. The choice of carrier layer material can also affect other areas of actuator operation [21]. 

Nowadays, research on control systems of cantilever-type piezoelectric actuators focuses mainly on the compensation of nonlinear phenomena: hysteresis [22] and/or creep [23]. Mathematical models of the aforementioned nonlinear phenomena, proposed by the authors, expand a linear model, which can be lumped [24] or continuous [25]. Continuous models are the direct basis for prototyping control laws, which use state space: LQR [26] and LQG [27]. In published research results, a continuous model is usually constructed for only one selected material of the carrier layer. It should be noted that the influence of the difference between the Young’s modulus of the carrier layer and the piezoelectric layer is considered in energy harvesting models [28].

There are no research results presented in the available literature regarding the influence of the material of the carrier layer on the selection of weights in linear LQR control. Most often, these weights are selected by the trial-and-error method for one selected carrier material. Among the few other methods for one selected carrier material, the following can be distinguished: Ebrahimi-Tirtashi et al. [25] used Bryson’s rule; Wang et al. [26] noticed that the initial values of the weights should be chosen as the desired maximum squared values under the steady states and inputs; Tian et al. [29] proposed a genetic algorithm for weights selection. In this article, an experimental analysis of the impact of the carrier layer material on actuator performance was carried out. Based on the results of laboratory experiments, a modification of Bryson’s rule of weights selection in matrix R was proposed. The modification enabled effective control regardless of the material of the supporting layer, the maximum set value of the actuator displacement, and the duration of this set value at a constant level.

## 2. Materials and Methods

### 2.1. Materials 

**Figure 1:**
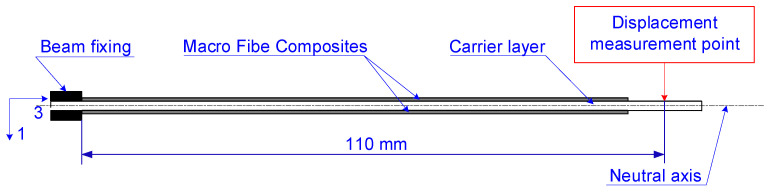
Schema of a cross-section of a cantilever beams: 1—longitudinal axis of the beam, 3—transverse axis of the beam.

**Figure 2:**
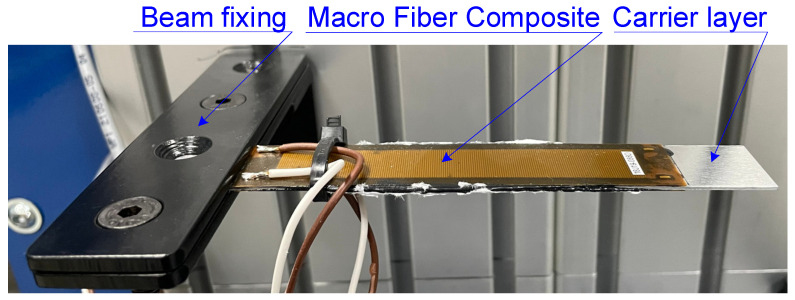
Produced cantilever beam containing aluminum carrier layer.

Two manufactured piezoelectric cantilever sandwich beams were the research objects. The beams differed in the material of the carrier layer. In the first beam, the carrier layer was made of glass-reinforced epoxy composite (FR4), produced by W.P.P.H.U. HATRON S.C., Kraków, Poland, and in the second beam, it was made of 1050 aluminum. The schema of the cross-section of both cantilever beams is shown in Figure 1 and a view of one of the produced cantilever beams in Figure 2.

Each cantilever beam consisted of one carrier layer and two piezoelectric layers. Patches of macro fiber composite (MFC) type P1 [30], produced by Smart Material Corp., Sarasota, FL, USA, were used as piezoelectric layers. The MFC patches were symmetrically glued to both sides of the carrier layer. Epoxy adhesive DP490 [31], produced by the 3M company, Saint Paul, MN, USA, was used to create a glued connection between the MFC patches and the carrier layer. The geometric properties of the manufactured cantilever are presented in Table 1.

### 2.2. Laboratory Research Method 

**Figure 3:**
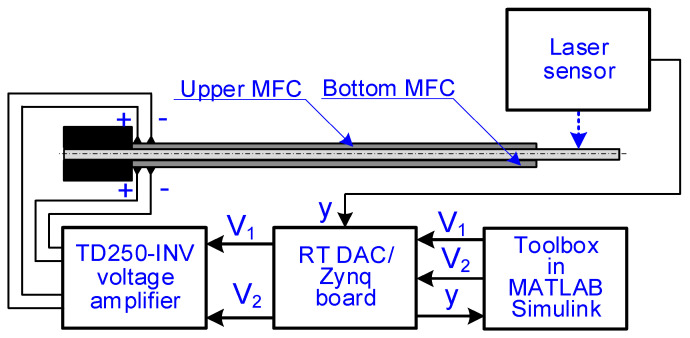
Schema of measurement system.

The motion of the beam was forced by using a system consisting of a computer with MATLAB Simulink software, an A/D board, and a voltage amplifier. The generation of control voltage waveforms, which were supplied to the MFC patch/patches, was performed in the 2019b version of the MATLAB Simulink program, in which the solver ode1 was used to perform the calculations. A fixed-step equal to 0.001 s was used in these calculations to obtain real-time calculations. The generated voltage waveforms were sent to the TD250-INV voltage amplifier, produced by PiezoDrive company, Shortland, Australia, in real time. The voltages were sent in real time using an RT_DAC/Zynq A/D board, manufactured by INTECO company Kraków, Poland, integrated with a dedicated MATLAB toolbox described in [32]. The TD250-INV voltage amplifier generated from one to two control voltages in the range from −500 V to +500 V. In all laboratory experiments, the displacement of one point in the cantilever beam structure was measured. The distance between the measured point and the beam fixing was 110 mm. The measurement system contained an LG5B65PI laser sensor of displacement, produced by BANNER company, Minneapolis, MN, USA, and the aforementioned RT_DAC/Zynq A/D board, which enabled data acquisition in real time. The LG5B65PI laser sensor had a measurement resolution equal to 40 microns for the measurement at a frequency equal to 450 Hz, and the analog linearity was ±10 microns. The measurement system schema is shown in Figure 3. 

The actuator temperature was measured using the Flir E40 thermovision camera during the longest experiments, which lasted 26 s. No observable temperature changes were noted between the beginning and end of the experiment. Temperature changes affect the electrical impedance of the piezoelectric layer [33,34], but the position error of the actuator resulting from warm-up only appears where the actuator is excited for a long time, and even then, this error is very small [35].

### 2.3. Simulation Research Method 

Simulation experiments were carried out in the 2019b version of the MATLAB Simulink program, in which the solver ode23tb was used to perform the calculations. A variable-step was used in these calculations. The ode23tb algorithm is an implementation of the TR-BDF2 method, which is a combination of trapezoidal and second-order backward differentiation [36]. The purpose of the simulations was to determine the displacement of the tip of the cantilever beam caused by an applied control voltage, of which the values were assumed in advance, to one (unimorph) or two MFC patches (bimorph). A variable-step was used in the simulation research because only such a step enabled the simulation of the operation of the actuator described by a mathematical model containing matrixes of very large sizes. The simulations did not attempt to obtain the real-time response of the modeled system.

## 3. Mathematical Model of Piezoelectric Actuator and Synthesis of Control System

### 3.1. Piezoelectric Actuator

The displacements of selected points in the cantilever beam structure were calculated using a mathematical model, which was built on the basis of two methods: Finite Element Method (FEM) and State Space Method. FEM was used because a tip mass does not occur [37]. The mathematical model was built in two stages: (1) determination of stiffness matrix **K_g_** and mass matrix **M_g_** for the assumed number of finite elements; and (2) determination of state matrix **A**, control matrix **B**, output matrix **C**, and feed-through matrix **D**. 

The structure of the cantilever beam, which is shown in Figure 1, was divided into 48 finite elements, each of a length equal to 2.5 mm. As a result of this division, 49 nodes were created. A total of 48 nodes had two degrees of freedom, and one node, which was in the beam fixing, had zero degrees of freedom. A motion equation can be given by [25]:

(1) $$M_{g}\overset{••}{d(t)}+C_{g}\overset{•}{d(t)}+K_{g}d(t)={Ε}_{1}V_{1}(t)+E_{2}V_{2}(t)$$

where **M_g_** is a global mass matrix (dimensions: 96 × 96 for 48 nodes), **C_g_** is a global damping matrix (dimensions: 96 × 96 for 48 nodes), **K_g_** is a global stiffness matrix (dimensions: 96 × 96 for 48 nodes), **E_1_** (dimensions: 96 × 1 for 48 nodes) and **E_2_** (dimensions: 96 × 1 for 48 nodes) are localization matrixes of forces generated by the upper MFC patch and the bottom MFC patch, V_1_ and V_2_ are voltages applied to the upper MFC patch and the bottom MFC patch, and **d** is a vector of vertical (w) and rotational (φ) displacements: **d** = [w, φ]^T^ of node. Local mass matrixes **M_l_** and local stiffness matrixes **K_l_** were calculated as follows [26]:

(2) $$M_{l}=M_{lc}+2M_{lmfc}\;\;\;{K_{l}=K}_{lc}+2K_{lmfc}$$

where **M**_lc_ and **K**_lc_ are local mass and stiffness matrixes of the carrier layer, and **M**_lmfc_ and **K**_lmfc_ are the local mass and stiffness matrixes of MFC:

(3) $$M_{lc}=\frac{\rho_{c}A_{c}l_{e}}{420}\left[\begin{array}{cccc} 156 & 22l_{e} & 54 & -13l_{e}\\ 22l_{e} & 4l_{e}^{2} & 13l_{e} & -3l_{e}^{2}\\ 54 & 13l_{e} & 156 & -22l_{e}\\ -13l_{e} & -3l_{e}^{2} & -22l_{e} & 4l_{e}^{2} \end{array}\right]\;K_{lc}=\frac{E_{c}\eta I_{c}}{l_{e}}\left[\begin{array}{cccc} 12 & 6l_{e} & -12 & 6l_{e}\\ 6l_{e} & 4l_{e}^{2} & -6l_{e} & 2l_{e}^{2}\\ -12 & -6l_{e} & 12 & -6l_{e}\\ 6l_{e} & 2l_{e}^{2} & -6l_{e} & 4l_{e}^{2} \end{array}\right]\;M_{lmfc}=\frac{\rho_{\mathrm{mfc}}A_{mfc}l_{e}}{420}\left[\begin{array}{cccc} 156 & 22l_{e} & 54 & -13l_{e}\\ 22l_{e} & 4l_{e}^{2} & 13l_{e} & -3l_{e}^{2}\\ 54 & 13l_{e} & 156 & -22l_{e}\\ -13l_{e} & -3l_{e}^{2} & -22l_{e} & 4l_{e}^{2} \end{array}\right]\;\;K_{lmfc}=\frac{E_{mfc}\eta I_{mfc}}{l_{e}}\left[\begin{array}{cccc} 12 & 6l_{e} & -12 & 6l_{e}\\ 6l_{e} & 4l_{e}^{2} & -6l_{e} & 2l_{e}^{2}\\ -12 & -6l_{e} & 12 & -6l_{e}\\ 6l_{e} & 2l_{e}^{2} & -6l_{e} & 4l_{e}^{2} \end{array}\right]$$

where ρ_c_ is the density of the carrier layer, A_c_ is the cross-section area of the carrier layer, l_e_ is the length of the finite element, E_c_ is Young’s modulus of the carrier layer, I_c_ is the moment of inertia of the carrier layer, ρ_mfc_ is the density of the MFC patch, A_mfc_ is the cross-section area of the MFC patch, E_mfc_ is Young’s modulus of the MFC patch, I_mfc_ is the moment of inertia of the MFC patch, and η is the ratio of the piezoelectric material elastic constant to that constant of the carrier layer material:

(4) $$\eta =\frac{Y_{mfc}}{Y_{c}}$$

A global damping matrix was calculated as proportional damping in the Rayleigh form [38]:

(5) $$C_{g}=\alpha M_{g}+\beta K_{g}$$

where α and β are the dimensionless coefficients, which were selected experimentally.

Considering that both the upper MFC and the bottom MFC are equidistant from the neutral axis of the cantilever beam, the bending moment can be calculated in the same way for both MFCs. The bending moment per unit length generated by each MFC patch in the vertical axis (axis 1 in Figure 1) is calculated as follows:

(6) $$M_{\mathrm{bi}}\left(t\right)=\gamma \int_{\frac{1}{2}t_{cr}}^{\frac{1}{2}t_{cr}+t_{\mathrm{mfca}}}d_{33a}Y_{33a}\frac{V_{i}\left(t\right)}{t_{\mathrm{mfce}}}w_{\mathrm{mfca}}y\mathrm{dy}=\frac{1}{2}\gamma d_{33a}Y_{\mathrm{mfca}}\frac{V_{i}\left(t\right)}{t_{\mathrm{mfce}}}w_{\mathrm{mfca}}\left(t_{cr}t_{\mathrm{mfca}}+t_{\mathrm{mfca}}^{2}\right)$$

where γ is the ratio of the smaller Young’s modulus to the larger one for a pair of two materials (the active part of the MFC patch and the carrier layer (ratio Y_c_/Y_mfca_ for the composite carrier layer and Y_mfca_/Y_c_ for the aluminum carrier layer)), and t_cr_ is the carrier layer thickness increased by half the thickness of the passive layer in the MFC patch. It was assumed that an equivalent concentrated force is applied, which generates the value of the bending moment calculated according to (6), at the center of gravity of each MFC patch. Therefore, the equivalent concentrated force generated by the MFC patch in the direction of axis 1, acting on the cantilever beam, can be given by

(7) $$P_{i}(t)=\frac{M_{bi}(t)}{{0.5(l}_{mfca}+l_{mfcp})}$$

The point of application of the equivalent concentrated force was also assumed at the center of gravity of the MFC patch, which is located at the 20th node (distance 50 mm from beam fixing). The matrixes of forces localization for the upper MFC (**E_1_**) and the bottom MFC (**E_2_**) [39] are calculated as follows:

(8) $$E_{1}={\frac{1}{2}{\gamma d}_{33a}Y_{\mathrm{mfca}}\frac{1}{t_{\mathrm{mfce}}}w_{\mathrm{mfca}}\left(t_{cr}t_{\mathrm{mfca}}+t_{\mathrm{mfca}}^{2}\right)\left[\begin{array}{ccc} \Theta_{1\times 38} & \varepsilon_{1} & \Theta_{1\times 57} \end{array}\right]}^{T}\;E_{2}=\frac{1}{2}{\gamma d}_{33a}Y_{\mathrm{mfca}}\frac{1}{t_{\mathrm{mfce}}}w_{\mathrm{mfca}}\left(t_{cr}t_{\mathrm{mfca}}+t_{\mathrm{mfca}}^{2}\right){\left[\begin{array}{ccc} \Theta_{1\times 38} & {-\varepsilon }_{2} & \Theta_{1\times 57} \end{array}\right]}^{T}$$

where ε_1_ is a coefficient showing the contribution of the stretching MFC in generating the motion of the cantilever beam (it was assumed that the value of this parameter will be 1 in simulation studies), and ε_2_ is a coefficient showing the contribution of the compressing MFC in generating the motion of the cantilever beam. It was determined in laboratory experiments that for the composite-based actuator ε_1_ = 1 and ε_2_ = 0.36, and for the aluminum-based actuator ε_1_ = 1 and ε_2_ = 0.38. The material properties used in the simulation tests are presented in Table 2. 

Matrixes of forces localization were used to build a state space model, which had a well-known form:

(9) $$\overset{•}{x(t)}=Ax(t)+Bu(t)\;y(t)=Cx(t)+Du(t)$$

where **x** is a state vector (containing 192 state variables), **u** is an input vector, and **y** is an output vector. The matrix dimensions are as follows:

(10) $$x={\left[\begin{array}{c} d\\ \dot{d} \end{array}\right]}_{192\times 1}A={\left[\begin{array}{cc} \Theta_{96\times 96} & I_{96\times 96}\\ -M_{g}^{-1}K_{g} & -M_{g}^{-1}C_{g} \end{array}\right]}_{192\times 192}B={\left[\begin{array}{cc} \Theta_{96\times 1} & \Theta_{96\times 1}\\ M_{g}^{-1}E_{1} & M_{g}^{-1}E_{2} \end{array}\right]}_{192\times 2}\;C={\left[\begin{array}{ccc} \Theta_{1\times 86} & 1 & \Theta_{1\times 105} \end{array}\right]}_{1\times 192}D={\left[\begin{array}{cc} 0 & 0 \end{array}\right]}_{1\times 2}$$

An output variable was the 87th state variable, which was a displacement of the 44th node in the cantilever beam structure in the direction of axis 1. The 44th node was located 110 mm from the beam fixing. 

Taking into account that measurement data from the laboratory stand are available, an alternative method of modeling the actuator could be data-driven modeling [41].

### 3.2. Synthesis of Control System

A Linear Quadratic Gaussian (LQG) algorithm with integral feedback was used to generate two independent control voltages. The LQG consisted of a linear quadratic regulator (LQR) and a Kalman filter used to estimate the state vector. This algorithm has been extended with integral feedback. A synthesis of the control algorithm was based on the state space model (10). The basic condition for the implementation of the LQR algorithm is full controllability of the controlled object. The actuator described by (10) is fully controllable because there is at least one non-zero element in each row of a controllability matrix **Q_ctrb_**:

(11) $$Q_{ctrb}=\Phi^{-1}B$$

where Φ is the truncated matrix consisting of n eigenvectors. The actuator model in state space (10) was extended by the additional state variable, which is the integral of the difference between the set value and the measured value of the beam tip displacement:

(12) $$\begin{array}{c} \left[\begin{array}{c} \dot{x}(t)\\ {\dot{x}}_{n+1}(t) \end{array}\right]=\left[\begin{array}{cc} A & \Theta_{192\times 1}\\ -C & 0 \end{array}\right]\left[\begin{array}{c} x(t)\\ x_{n+1}(t) \end{array}\right]+\left[\begin{array}{c} B\\ 0 \end{array}\right]u(t)\\ y(t)=\left[\begin{array}{cc} C & 0 \end{array}\right]\left[\begin{array}{c} x(t)\\ x_{n+1}(t) \end{array}\right]+Du(t) \end{array}$$

The basic condition for implementation of the Kalman filter is full observability of the controlled object. The actuator described by (10) is fully observable because there is at least one non-zero element in each column of the observability matrix **Q_obsv_**:

(13) $$Q_{obsv}=C\Phi$$

The estimated state vector based on the Kalman filter is

(14) $${\dot{x}}_{e\mathrm{st}}(t)=Ax_{est}(t)+Bu(t)+H\left(y_{measured}(t)-Cx_{est}(t)\right)-HDu(t)$$

where **x**_est_ is the estimated state vector and **H** is the gains matrix:

(15) $$H=PC^{T}{R_{c}}^{-1}$$

where **R**_c_ is the covariance matrix of measurement noise and **P** is the solution of the algebraic Ricatti equation:

(16) $$AP+PA^{T}-PC^{T}{R_{c}}^{-1}CP^{T}+Q_{c}=0$$

where **Q**_c_ is the covariance matrix of state noise.

**Figure 4:**
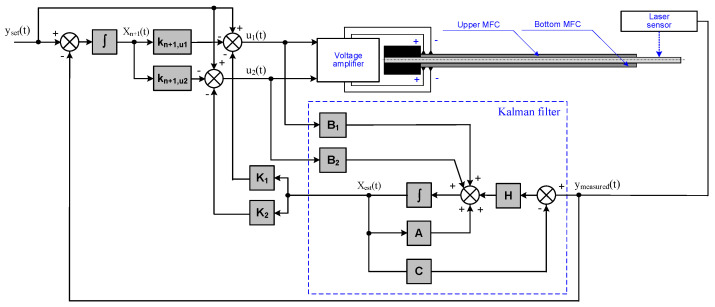
Schema of control system: B_1_—first column of B matrix, B_2_—second column of B matrix.

The final control law considering the estimated state vector is

(17) $$u_{1}\left(t\right)=-\left[\begin{array}{cc} K_{1} & k_{n+1,u_{1}} \end{array}\right]\left[\begin{array}{c} x_{est}(t)\\ x_{n+1}(t) \end{array}\right]+y_{set}\left(t\right)\;u_{2}(t)=-\left[\begin{array}{cc} K_{2} & k_{n+1,u_{2}} \end{array}\right]\left[\begin{array}{c} x_{est}(t)\\ x_{n+1}(t) \end{array}\right]+y_{set}(t)$$

where n is the size of the state vector, **K_1_** and **K_2_** are the matrixes of the state variable gains for u_1_ and u_2_, respectively, k_n+1,u1_ and k_n+1,u2_ are the gains in the integral feedback for u_1_ and u_2_, respectively, and y_set_ is the set value of the actuator tip displacement. The gains **K_1_**, **K_2,_** k_n+1_,_u1,_ k_n+1_,_u2_ were calculated by the minimization of the expanded quality index:

(18) $$J=\int_{0}^{\infty }\left({\left[\begin{array}{c} x(t)\\ x_{n+1}(t) \end{array}\right]}^{T}Q\left[\begin{array}{c} x(t)\\ x_{n+1}(t) \end{array}\right]+u^{T}(t)Ru(t)\right)\mathrm{dt}$$

where **Q** is the positive definite or semi-definite weight matrix, and **R** is the positive definite weight matrix. The measurement system schema is shown in Figure 4.

## 4. Results

Laboratory research included experiments in which the step responses of the unimorph and the bimorph with both a composite and an aluminum carrier layer were measured. The research was divided into two stages: (1) laboratory and simulation research regarding the impact of the carrier layer material on actuator performance and (2) laboratory research regarding the control system of the actuator.

### 4.1. Description of First Stage of Research

The first stage of research included a determination of the duration of the transition period in the creep process and a determination of the impact of the carrier layer material on actuator performance. To determine the duration of the transition period in the creep process, step responses were measured. The measurement was performed for the spike of voltage V_1_ or simultaneous spikes of voltages V_1_ and V_2_ from 0 to the set value. The spike in voltage or voltages started in the first second and lasted for 2 s. The experiment conditions for both composite-based and aluminum-based actuators are presented in Table 3.

**Figure 5:**
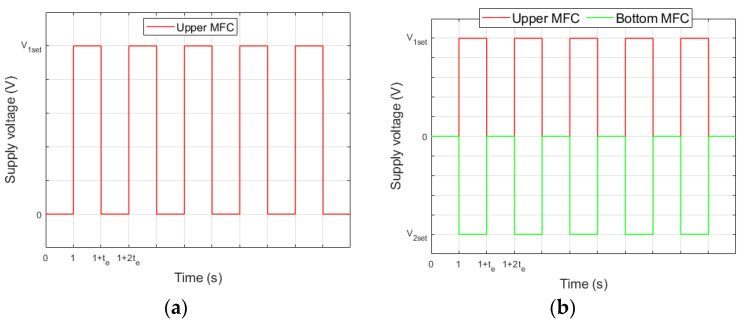
Supply voltage waveforms in laboratory research: (**a**) unimorph, (**b**) bimorph.

To determine the impact of carrier layer material on actuator performance, the supply voltage of the upper MFC patch was increased from 0 to the set value (both for unimorph and bimorph) and the simultaneous supply voltage of the bottom MFC patch was decreased from 0 to the set value (only for bimorph). The supply voltage waveforms are shown in Figure 5 (t_e_ is the duration time of the voltage spike). It should be noted that the upper MFC generated tensile stresses above the neutral axis (Figure 1) in the cantilever beam in both the unimorph and the bimorph. In contrast, the bottom MFC generated compressive stresses below the neutral axis in the bimorph.

Five spikes of supply voltage V_1_ for the unimorph as well as five spikes of V_1_ and simultaneous V_2_ for the bimorph were generated. The experiment conditions for both composite-based and aluminum-based actuators are presented in Table 4.

### 4.2. Results in First Stage of Research

**Figure 6:**
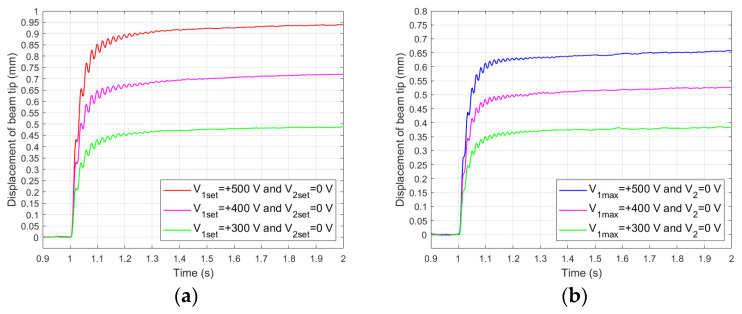

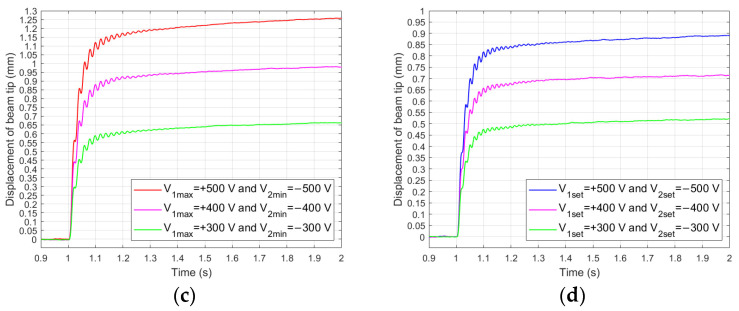
Step responses: (**a**) composite-based unimorph, (**b**) aluminum-based unimorph, (**c**) composite-based bimorph, (**d**) aluminum-based bimorph.

Figure 6 shows the comparison of step responses obtained in laboratory experiments for both the composite and the aluminum carrier layer. In general, the duration of the transition periods is approximately the same for both the unimorph and the bimorph, as well as for the composite and aluminum carrier layers. It can be assumed that the duration of the transition periods does not exceed 0.3 s (from 1 to 1.3 s).

**Figure 7:**
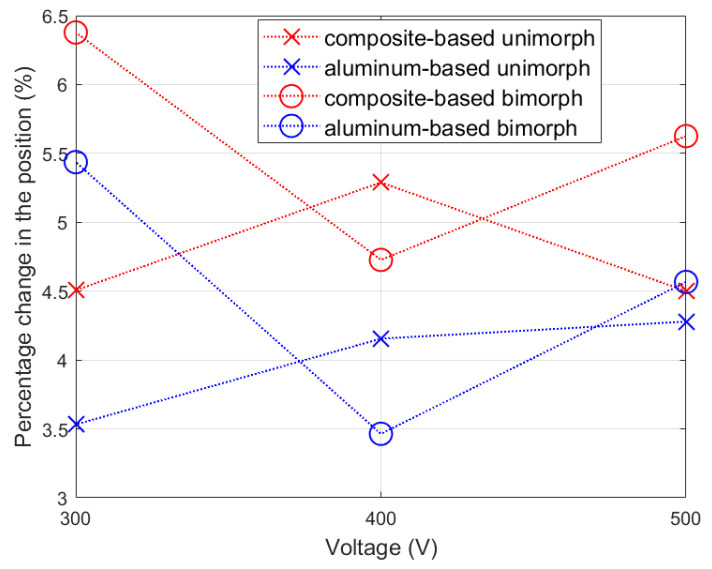
Percentage change in the position of the cantilever beam tip caused by the creep process.

The creep process itself, however, varied depending on whether there was a composite or aluminum carrier layer. The percentage changes in the beam tip displacement in time from 1.3 s to 2 s are shown in Figure 7.

The actuator containing a composite carrier layer exhibited significantly larger creep-induced displacements in comparison to the actuator containing an aluminum carrier layer. 

**Figure 8:**
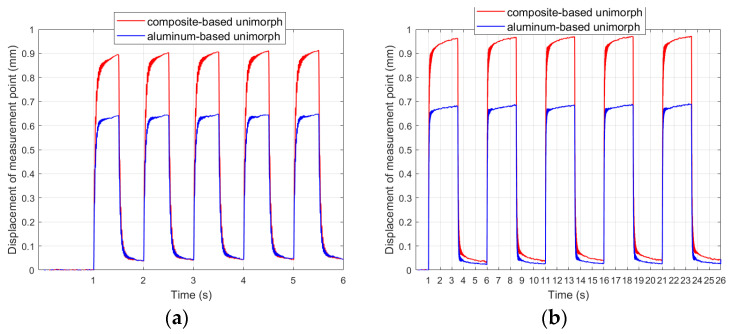
Step responses of unimorph for V_1set_ = +500 V: (**a**) t_e_ = 0.5 s, (**b**) t_e_ = 2.5 s.

Figure 8 shows the comparison of results obtained in laboratory experiments no. 7 and no. 11 for both composite-based and aluminum-based actuators. 

**Figure 9:**
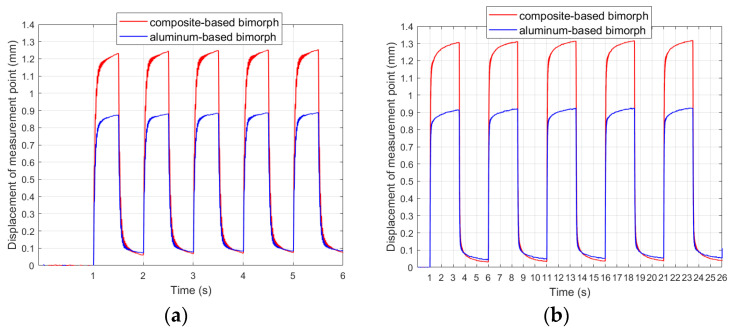
Step responses of bimorphs for V_1set_ = +500 V and V_2set_ = −500 V: (**a**) t_e_ = 0.5 s, (**b**) t_e_ = 2.5 s.

The first observation was that there were larger displacements of the composite-based actuator compared to the aluminum-based actuator, which is consistent with the observations of other researchers regarding the influence of stiffness on the achieved displacements [18]. In experiment no. 7, the average displacement of the actuator containing an aluminum carrier layer was 70.3% of the displacement of the actuator containing a composite carrier layer, and it was 70.7% in experiment no. 11. Figure 9 shows the comparison of the results obtained in laboratory experiments no. 22 and no. 26 for bimorphs containing a composite or aluminum carrier layer. 

The composite-based actuator achieved larger displacements than the aluminum-based actuator. This difference was approximately constant for different time durations of the applied voltage spike. The average displacement of the aluminum-based actuator was 70.7% of the displacement of the composite-based actuator in experiment no. 22 and was 68.8% in experiment no. 26. On this basis, the ε_2_ coefficient, which is needed in the mathematical model (Section 3.1), was determined: ε_2com_ = 0.367 for the composite carrier layer and ε_2alu_ = 0.388 for the aluminum carrier layer.

**Figure 10:**
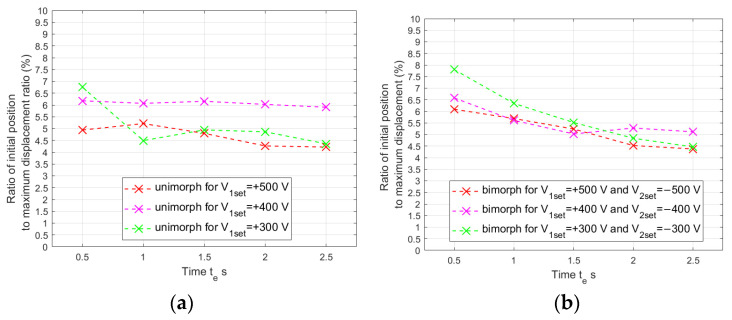
Ratio of initial position to maximum displacement of composite-based actuator: (**a**) unimorph, (**b**) bimorph.

In Figure 8 and Figure 9 it can be noticed that the actuator does not return to its initial position after the voltage spike stops. This phenomenon occurs regardless of the voltage value and the duration of the voltage spike. This is due to the phenomenon of hysteresis. Figure 10 shows the ratios of the initial positions of the composite-based actuators to the maximum displacement of these actuators.

**Figure 11:**
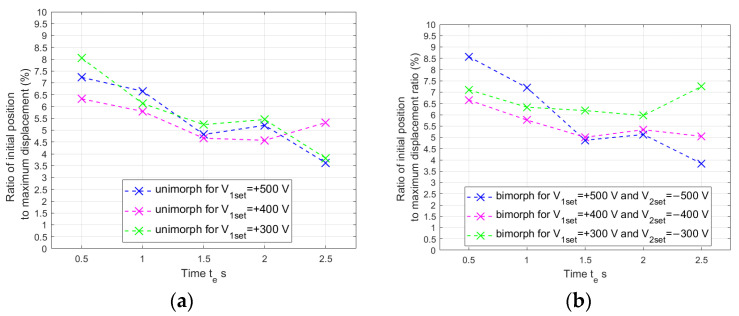
Ratio of initial position to maximum displacement of aluminum-based actuator: (**a**) unimorph, (**b**) bimorph.

In general, the position in the interval among voltage spikes (initial position) becomes a smaller and smaller part of the maximum actuator displacement as the duration of the voltage spike increases. Therefore, it can be concluded that changes in the initial position occur at a slightly slower rate than changes in the maximum position of the actuator. The initial position is, on average, from 4.69% to 6.07% of the maximum position in the case of the unimorph and from 5.18% to 5.79% in the case of the bimorph. It can be assumed that the initial position before the next voltage spike is linearly proportional to the maximum displacement of the actuator caused by the previous voltage spike. On this basis, the values of the new coefficient θ were determined for each condition, which specify linear correction of the simulated voltage values applied to the upper and bottom MFCs in the intervals between voltage spikes compared to laboratory values: for the unimorph, instead of V_1_ = 0, it should be V_1_= θV_1set_, and for the bimorph, instead of V_1_ = 0, it should be V_1_= θV_1set_ and instead of V_2_ = 0, it should be V_2_= θV_2set_. A similar analysis was performed for the actuator that contains an aluminum carrier layer (Figure 11). Also, for such actuators, the initial position is an approximately constant part of the maximum position. The initial position is, on average, from 5.35% to 5.75% of the maximum position in the case of the unimorph, and from 5.94% to 6.57% in the case of the bimorph. Similarly, for the composite layer, the initial position before the next voltage spike is linearly proportional to the maximum displacement of the actuator caused by the previous voltage spike. On this basis, the coefficient values of coefficient θ were determined for each condition, which specify linear correction of the simulated voltage values applied to the upper and bottom MFCs in the intervals between voltage spikes compared to laboratory values: for the unimorph, instead of V_1_ = 0, it should be V_1_= θV_1set_, and for the bimorph, instead of V_1_ = 0, it should be V_1_ = θV_1set_ and instead of V_2_ = 0, it should be V_2_ = θV_2set_. 

**Figure 12:**
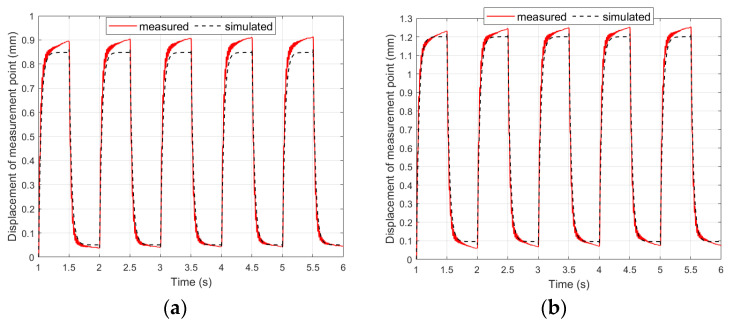
Comparison of simulation results and laboratory results for composite-based actuator: (**a**) unimorph, (**b**) bimorph.

**Figure 13:**
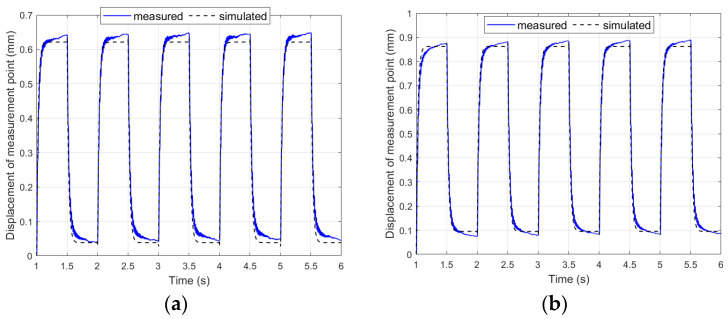
Comparison of simulation results and laboratory results for aluminum-based actuator: (**a**) unimorph, (**b**) bimorph.

To obtain simulation results consistent with the laboratory results, two more significant corrections were introduced to the linear mathematical model in comparison to models known from the literature. The first of these corrections was to consider the difference between the Young’s modulus of the piezoelectric material and the Young’s modulus of the carrier layer material. The value of the generated bending moment depends on the ratio between these Young’s moduli. This relationship was introduced by using the γ coefficient in (8). This coefficient made it possible to adapt the linear model to the materials of the carrier layer, which differ in the value of Young’s modulus. The second correction also concerned the generation of the bending moment: the thickness of only the piezoelectric fiber in the MFC patch was used in the model. Other researchers have used the thickness of the whole MFC patch [42] or half the thickness of the whole MFC patch [43]. A comparison of the results obtained in laboratory tests with the simulation results obtained on the basis of the modified linear model presented in Section 3.1 is shown in Figure 12 and Figure 13.

The introduction of the first correction to the mathematical model makes it possible to adapt this model to various materials of the carrier layer. On the basis of the research, it was noticed that compliance of the simulation results with laboratory results, for the same model in the state space but for different materials of the carrier layer, can be achieved through this correction of the bending moment calculation. The introduction of the second correction allows the calculation of the bending moment, which is more consistent with the generated bending moment in the actuator beam.

### 4.3. Description of Second Stage of Research

The second stage of research included a determination of the impact of the material properties of the carrier layer on the weights in the quality index in the LQG control algorithm. To reduce the computational cost in the control system in the laboratory stand, the model in state space (10) was reduced to the first mode. For this purpose, the nodal displacements vector was transformed into a reduced vector:

(19) $$d=\Phi_{m}\kappa$$

where **Φ_m_** is the truncated matrix and κ is the modal coordinate vector. The modal matrixes for first mode are as follows:

(20) $$K_{gm}=\Phi_{m1}^{T}K_{g}\Phi_{m1}M_{gm}=\Phi_{m1}^{T}M_{g}\Phi_{m1}C_{gm}=\Phi_{m1}^{T}C_{g}\Phi_{m1}\;E_{1m}=\Phi_{m1}^{T}E_{1}E_{2m}=\Phi_{m1}^{T}$$

where **Φ_m1_** is the truncated matrix for the first mode. The model in the state space for the first mode is as follows:

(21) $$x_{m}={\left[\begin{array}{c} \kappa \\ \dot{\kappa } \end{array}\right]}_{2\times 1}A_{m}={\left[\begin{array}{cc} 0 & 1\\ -M_{gm}^{-1}K_{gm} & -M_{gm}^{-1}C_{gm} \end{array}\right]}_{4\times 4}B_{m}={\left[\begin{array}{cc} 0 & 0\\ M_{gm}^{-1}E_{1m} & M_{gm}^{-1}E_{2m} \end{array}\right]}_{4\times 2}\;C_{m}=\left[\begin{array}{cc} \varphi_{87} & 0 \end{array}\right]D_{m}=\left[\begin{array}{cc} 0 & 0 \end{array}\right]$$

where φ_87_ is 87th element of the truncated matrix **Φ_m_**. The matrixes **A_m_**, **B_m_**, **C_m_**, and **D_m_** were introduced to Equations (12) and (14)–(18) in the laboratory research.

**Figure 14:**
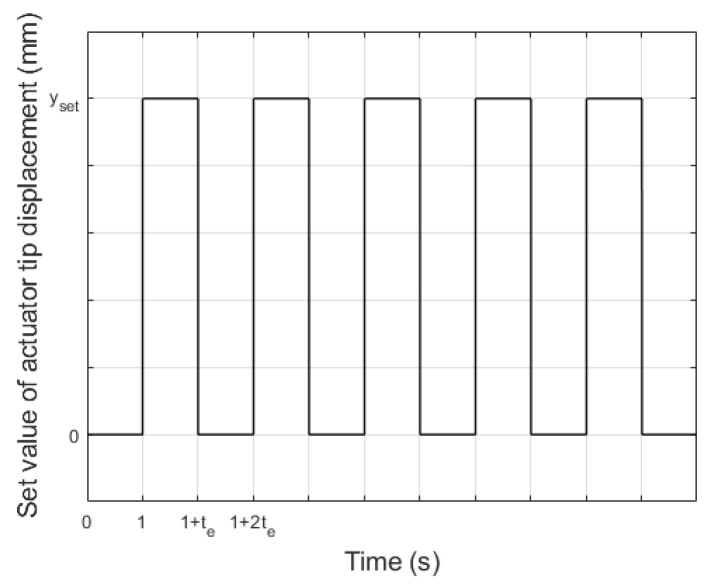
Waveforms of set value of actuator tip displacement for five spikes.

The waveforms of the set value of the actuator tip displacement are shown in Figure 14 (t_e_ is the duration time of set value spike) for both the unimorph and bimorph actuators.

The first problem was to determine the set value of the actuator displacement (y_set_) that can be achieved for the maximum (minimum) value of the control voltage without occurrence of displacement caused by the creep phenomenon. The hardware conditions, which are described in Section 2.2, showed that the maximum and minimum control voltage values were +500 V and −500 V, respectively. Values of y_set_ corresponding to ±500 V were determined experimentally based on the laboratory results, which are presented in Figure 6. On the basis of results presented in Figure 6, y_set_ values corresponding to ±400 V and ±300 V were also read. In this way, three values of y_set_ were established. In addition to these, one additional smaller value of y_set_ was established. The experiment conditions are presented in Table 5.

In the mathematical model (6–10) that was used to prototype control voltages u_1_ and u_2_, the values of the coefficients γ, ε_1_, ε_2com_ and ε_2alu_ were equal to 1. 

### 4.4. Results in Second Stage of Research

Considering Equations (18)–(21), the **R** matrix has the following form:

(22) $$For\;unimorph:R=\left[r_{11}\right]\;\;\;For\;bimorph:R=\left[\begin{array}{cc} r_{11} & 0\\ 0 & r_{22} \end{array}\right]\;$$

**Figure 15:**
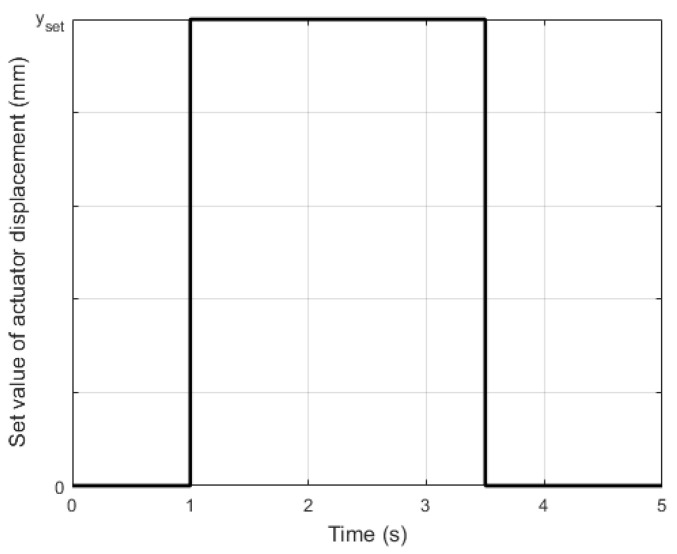
Waveforms of set value of actuator tip displacement for one spike.

Bryson’s rule was adopted as the basis for the selection of weights. Taking into account the γ coefficient introduced in Equation (8), an analysis of the impact of reducing the maximum control voltage on the control quality was carried out. The course of the set value is shown in Figure 15.

The set value of the actuator displacement was equal to 0.84 mm for the composite-based unimorph, 1.12 mm for the composite-based bimorph, 0.57 mm for the aluminum-based unimorph, and 0.85 mm for the aluminum-based bimorph. The first two weights in the Q matrix were selected based on [25], and the third weight was selected using the trial-and-error method:

(23) $$Q=\left[\begin{array}{ccc} 0.01 & 0 & 0\\ 0 & 0.01 & 0\\ 0 & 0 & 30.33\times 10^{9} \end{array}\right]\;$$

**Figure 16:**
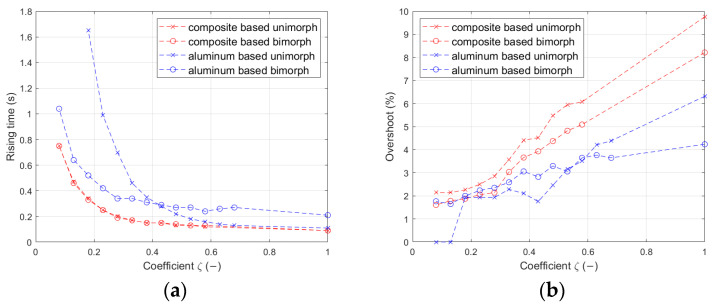
Impact of γ coefficient on quality indexes of control system: (**a**) on rising time, (**b**) on overshoot.

Figure 16 shows the impact of the value of the γ coefficient on the rising time and the overshoot of the control system output.

The rising time increased as the γ coefficient value decreased (Figure 16a). However, the overshoot increased as the γ coefficient value increased (Figure 16b). Hence, the choice of the γ coefficient value should be based on a compromise: on the one hand, the purpose should be to reduce the overshoot, and on the other hand, to shorten the rising time. Additionally, in the case of a bimorph, the weight in the **R** matrix that corresponds to the control voltage of the compressing MFC patch should be smaller than the weight corresponding to the control voltage of the stretching MFC patch. The following modification of Bryson’s rule is proposed:

(24) $$For\;composite\;based\;unimorph:r_{11}=\frac{1}{{\left|u_{1max}\right|}^{2}\gamma^{2}}\;For\;aluminum\;based\;unimorph:r_{11}=\frac{1}{{\left|u_{1max}\right|}^{2}\gamma^{2}}\;For\;composite\;based\;bimorph:r_{11}=\frac{\varepsilon_{1}}{{\left|u_{1max}\right|}^{2}\gamma^{2}}\;\;\;r_{22}=\frac{\varepsilon_{2}}{{\left|u_{2max}\right|}^{2}\gamma^{2}}\;For\;aluminum\;based\;bimorph:r_{11}=\frac{\varepsilon_{1}}{{\left|u_{1max}\right|}^{2}\gamma^{2}}\;\;\;r_{22}=\frac{\varepsilon_{2}}{{\left|u_{2max}\right|}^{2}\gamma^{2}}$$

The larger the value of the γ coefficient, the shorter the time it takes for the actuator to achieve the set displacement. A larger value of the γ coefficient can be used in actuator control systems with a carrier layer made of a stiffer material. This is due to the fact that the displacement caused by the creep phenomenon increases more slowly stiffer the material (compare Figure 7), which leads to a smaller increase in the overshoot. It was assumed that the γ coefficient for the composite-based actuator is equal to Y_s_/Y_mfca_ = 0.38 (Y_s_ means Young’s modulus of the FR4 composite) and that the γ coefficient for the aluminum-based actuator is equal to Y_mfca_/Y_s_ = 0.68 (Y_s_ means Young’s modulus of aluminum). On the basis of laboratory experiments in first stage of research, it was determined that for the composite-based actuator ε_1_ = 1 and ε_2_ = 0.36, and for the aluminum-based actuator ε_1_ = 1 and ε_2_ = 0.38. On the basis of the trial-and-error method, it was established that the weights in the **Q** matrix were equal to the largest value of the material constants that appear in Equation (2), which is Y_mfc_ for the composite-based actuator and Y_c_ for the aluminum-based actuator:

(25) $$For\;composite\;based\;actuator:Q=\left[\begin{array}{ccc} 30.33\times 10^{9} & 0 & 0\\ 0 & 0 & 0\\ 0 & 0 & 30.33\times 10^{9} \end{array}\right]\;\;For\;aluminum\;based\;actuator:Q=\left[\begin{array}{ccc} 71\times 10^{9} & 0 & 0\\ 0 & 0 & 0\\ 0 & 0 & 71\times 10^{9} \end{array}\right]\;$$

In all experiments, the weights in the **Q_c_** matrix with dimensions 3 × 3 and the **R_c_** matrix with dimensions 1 × 1, which are needed to calculate the **H** matrix in the Kalman filter, were the same (they were determined experimentally): **Q_c_** = diag(1 × 10^−3^, 1 × 10^−3^, 1 × 10^−3^) and **R_c_** = 1 × 10^−6^.

**Figure 17:**
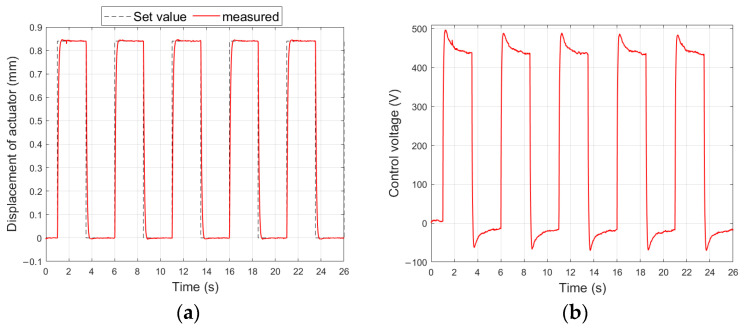

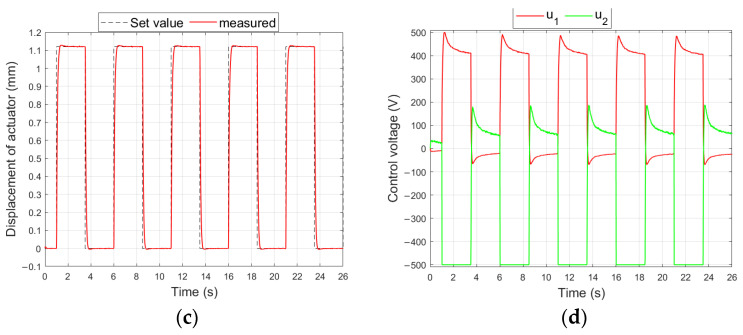
Control system characteristics for composite-based actuator: (**a**) unimorph displacement for y_set_ = 0.57 mm, (**b**) control voltage of unimorph, (**c**) bimorph displacement for y_set_ = 1.12 mm, (**d**) control voltages of bimorph.

Figure 17 shows the measured displacement of the composite-based actuator and the generated control signals waveforms, which were obtained in the control system shown in Figure 4 for the largest set values (experiments no. 37 and no. 53).

**Figure 18:**
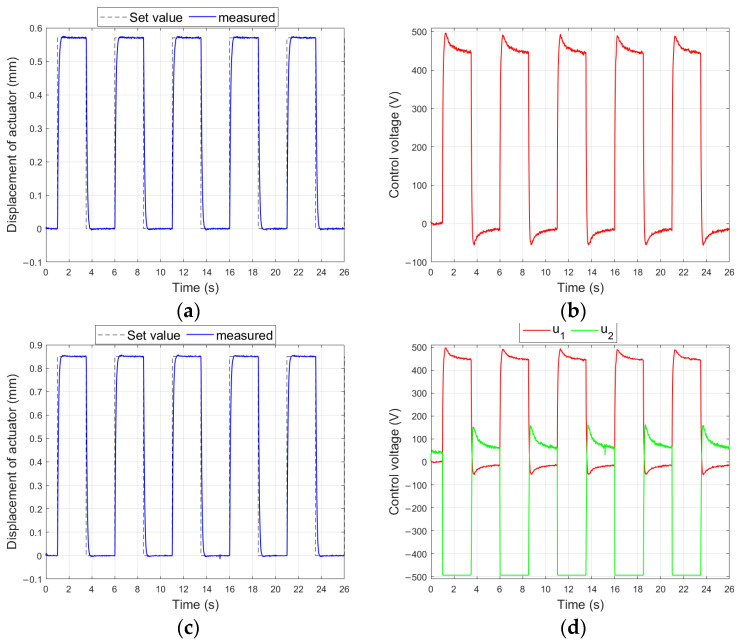
Control system characteristics for aluminum-based actuator: (**a**) unimorph displacement for y_set_ = 0.84 mm, (**b**) control voltage of unimorph, (**c**) bimorph displacement for y_set_ = 0.85 mm, (**d**) control voltages of bimorph.

Figure 18 shows the measured displacement of the aluminum-based actuator and the generated control signals waveforms, which were obtained in the control system shown in Figure 4 for the largest set values (experiments no. 39 and no. 55).

**Figure 19:**
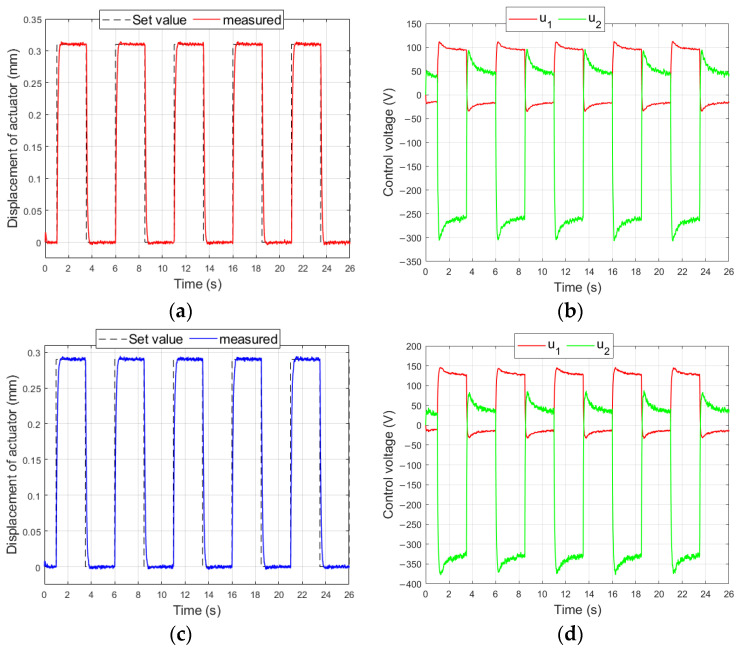
Control system characteristics for bimorph: (**a**) displacement of composite-based actuator for y_set_ = 0.31 mm, (**b**) control voltages of composite-based actuator, (**c**) displacement of composite-based actuator for y_set_ = 0.29 mm, (**d**) control voltages of composite-based actuator.

Figure 19 shows the characteristics which were obtained in the control system shown in Figure 4 for the smallest set values of bimorph displacement (experiments no. 65 and no. 67).

To compare the control quality in all 32 laboratory experiments (Table 5), a control quality index (I_q_) was determined in each of the experiments:

(26) $$I_{q}=\frac{1}{y_{setmax}}\int \left|y_{set}(t)-y(t)\right|\mathrm{dt}$$

where y_setmax_ is the maximum value of the set value of the actuator tip displacement. The I_q_ values for each experiment are presented in Table 6.

## 5. Discussion

**Figure 20:**
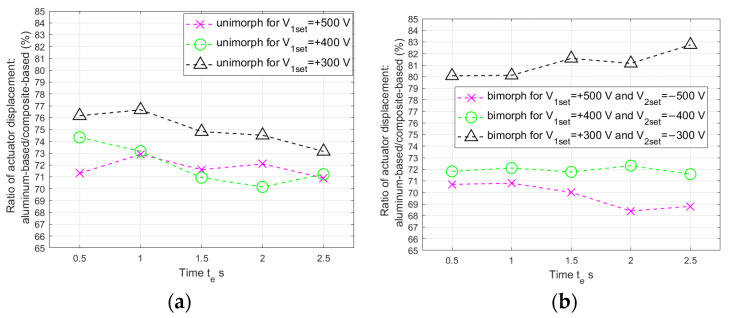
Aluminum-based actuator to composite-based actuator displacement ratio: (**a**) unimorph, (**b**) bimorph.

As expected, the displacements of the composite-based actuator appeared larger compared to the aluminum-based actuator, but this difference did not increase as the time duration of the applied voltage spike increased: these differences did not exceed 3% (Figure 20).

Therefore, it can be concluded that displacements caused by the creep phenomenon of the composite-based actuator were approximately proportional to displacements of the aluminum-based actuator. These displacements were proportionally larger in the case of the composite carrier layer in comparison to the aluminum carrier layer (Figure 6). 

**Figure 21:**
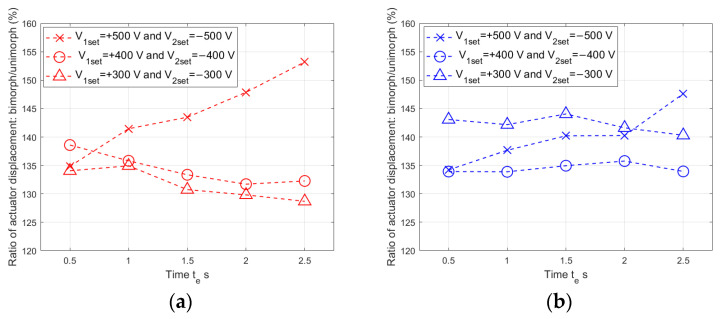
Bimorph actuator to unimorph actuator displacement ratio: (**a**) composite-based, (**b**) aluminum-based.

The ratios of bimorph to unimorph displacement are presented in Figure 21. 

It can be noticed that the difference between the bimorph and unimorph displacement increased for the largest values of voltage spikes (V_1set_ = +500 V and V_2set_ = −500 V), as the duration of the voltage spike increased: by 18.3% for the composite-based actuator and by 13.4% for the aluminum-based actuator. In the case of voltage spikes with other tested values (V_1set_ = +400 V and V_2set_ = −400 V, V_1set_ = +300 V and V_2set_ = −300 V), this difference decreased slightly as the duration of the voltage spike increased. The average displacement ratios were determined: with a composite carrier layer it was 136.72% and with an aluminum carrier layer it was 138.83%. 

Based on the results from the first stage of research, two main observations can be distinguished, which are important in the design of a linear control system of a piezoelectric actuator:

- The constant value of the control voltage causes undesirable actuator displacement, which is caused by the creep phenomenon. This is visible in Figure 6, Figure 8 and Figure 9;
- The control voltage of the compressing MFC should be larger than the control voltage of the stretching MFC. This observation is based on the comparison of the displacements of the unimorph and bimorph for the same carrier layer material.

These observations lead to guidelines for the determination of the weights in the **R** and **Q** matrixes:

- The use of Bryson’s rule to determine the weights in the **R** matrix is not sufficient because it leads to the generation of the maximum possible control voltage, for example ±500 V in the case of the equipment presented in this article. This article proposes a modification to the method of determining the weights by introducing the ratios of the Young’s modulus: see Equation (23). For the same purpose, in the **Q** matrix, the deviation from 0 of the first state variable should be limited by introducing an appropriately large weight q_11_. Based on the results of the laboratory experiments, the article proposes a weight value q_11_ equal to the larger value of Young’s modulus (either the Young’s modulus value of the carrier layer material or of the piezoelectric material);
- The weight in the **R** matrix that corresponds to the control voltage of the compressing MFC patch should be smaller than the weight corresponding to the control voltage of the stretching MFC patch. This article proposes a modification to the method of determining the weights by introducing the coefficient ε_2_: see Equation (24).

Based on the results of the first stage of research, it was also noted that the actuator positions in the intervals between control voltage spikes, which result from the hysteresis phenomenon, are approximately linearly dependent on the maximum displacement of the actuator. Reaching position zero in the intervals between control voltage spikes is possible by the application of a control voltage with the sign opposite to the sign of the voltage in the spikes. Obtaining position zero is possible by using a suitably large value of the weight q_33_ in the **Q** matrix. Based on the results of laboratory experiments, the article proposes a weight value q_33_ equal to the larger value of Young’s modulus (either the Young’s modulus value of the carrier layer material or of the piezoelectric material).

**Figure 22:**
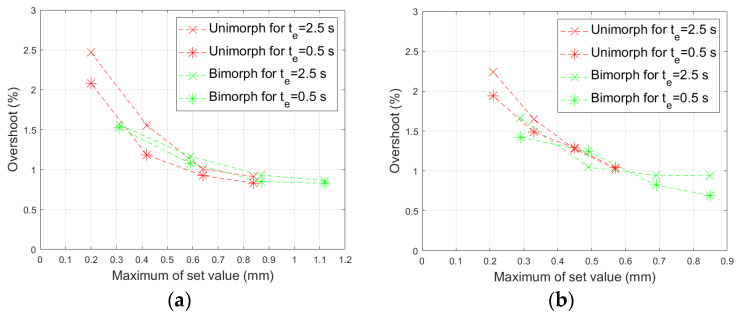
Overshoot: (**a**) composite-based actuators, (**b**) aluminum-based actuators.

The use of modified rules for determining weights in the **R** matrix together with experimentally selected weights in the **Q** matrix enabled effective linear control of actuators for both the composite and the aluminum carrier layers, and for different values of the set value of the actuator tip displacement. First of all, it was noticed that the actuator achieved y_set_ in each of the experiments whose conditions are given in Table 5. To compare the control quality in individual experiments, the overshoot value was calculated:

(27) $$\kappa =\left(\frac{y_{max}}{y_{steady}}100\%\right)-100\%$$

where y_max_ is the maximum value of the actuator tip displacement and y_steady_ is the actuator tip position in a steady state after reaching y_set_ (given in Table 5). A comparison of the overshoot values in the individual experiments is shown in Figure 22.

**Figure 23:**
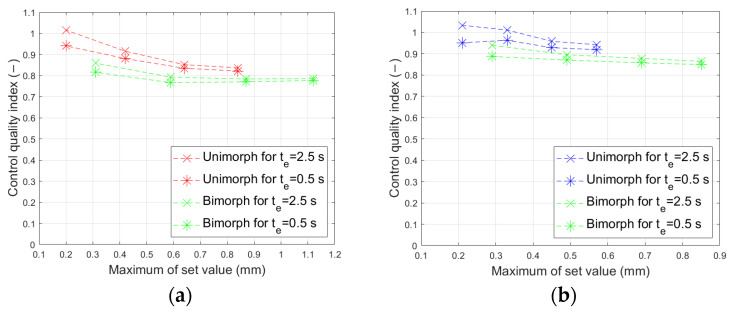
Quality index: (**a**) composite-based actuators, (**b**) aluminum-based actuators.

The overshoot value increased slightly as the maximum set value decreased. However, in no experiment did it exceed 2.5%. The range of the overshoot changes in the bimorph case is smaller than in the unimorph case. Figure 23 shows the comparison of the control quality index I_q_ (25) in all laboratory experiments.

As can be seen in Figure 21, the control quality is approximately similar regardless of the material of the carrier layer, the maximum of the set value, and the duration of this maximum.

## 6. Conclusions

The subject of this article was an experimental analysis of the control system of a composite-based piezoelectric actuator and an aluminum-based piezoelectric actuator. Analysis was performed for both the unimorph and bimorph structures. 

A modification of the method of selecting weights in the **R** matrix in the LQR control algorithm was proposed for a cantilever-type piezoelectric actuator. The weights in the R matrix for the actuator containing a carrier layer made of stiffer material should be smaller than those for the actuator containing a carrier layer made of less stiff material. Additionally, regardless of the carrier layer material, in the case of a bimorph, the weight in the **R** matrix that corresponds to the control voltage of the compressing MFC patch should be smaller than the weight corresponding to the control voltage of the stretching MFC patch.

The proposed correction of the selection of weights in the **R** matrix enables obtaining effective linear control, thanks to which displacements caused by the phenomenon of creep are eliminated. The quality of control remains approximately the same regardless of the material of the carrier layer, the maximum set value of the actuator displacement, and the duration of this set value at a constant level.

## Figures and Tables

**Table 1:** Dimensions of manufactured cantilever beams (in mm).

MFC Patch			Carrier Layer		
Dimension	Symbol	Value	Dimension	Symbol	Value
Total length	l_mfc_	100	Length	l_c_	120
Total width	w_mfc_	20	Width	w_c_	20
Total thickness	t_mfc_	0.3	Thickness	t_c_	1
Active part length	l_mfca_	85			
Active part width	w_mfca_	14			
Active part thickness	t_mfca_	0.18			
Passive part length	l_mfcp_	15			
Passive part thickness	t_mfcp_	0.12			
Distance between electrodes	t_mfce_	0.5			

**Table 2:** Material properties of manufactured cantilever beams.

Parameter		Composite-Based Actuator	Aluminum-Based Actuator
Young’s modulus of carrier layer	Y_c_	18.6 × 10^9^ Pa [38]	71 × 10^9^ Pa [26]
Density of carrier layer	ρ_c_	1850 kg/m^3^ [38]	2710 kg/m^3^ [26]
Young’s modulus of MFC patch	Y_mfc_	30.336 × 10^9^ Pa [30]	
Young’s modulus of MFC of piezoceramic fibers in MFC patch	Y_mfca_	48.3 × 10^9^ Pa [40]	
Density of active part of MFC patch	ρ_mfca_	5400 kg/m^3^ [26]	
Piezoelectric constant of MFC patch	d_33_	400 × 10^−12^ C/N [30]	
Piezoelectric constant of piezoceramic fibers in MFC patch	d_33a_	440 × 10^−12^ C/N [40]	

**Table 3:** Conditions of laboratory experiments to determine the duration of a transition period in creep process.

Experiment No.	1	2	3	4	5	6
Set voltage V_1set_ (V)	+500	+400	+300	+500	+400	+300
Set voltage V_2set_ (V)	0	0	0	−500	−400	−300

**Table 4:** Conditions of laboratory and simulation experiments to determine impact of carrier layer material on actuator performance.

	Experiment No.	V_1set_ (V)	V_2set_ (V)	t_e_ (s)				
Unimorph	7 to 11	+500	0	0.5	1	1.5	2	2.5
	12 to 16	+400	0	0.5	1	1.5	2	2.5
	17 to 21	+300	0	0.5	1	1.5	2	2.5
Bimorph	22 to 26	+500	−500	0.5	1	1.5	2	2.5
	27 to 31	+400	−400	0.5	1	1.5	2	2.5
	32 to 36	+300	−300	0.5	1	1.5	2	2.5

**Table 5:** Conditions of laboratory experiments in second stage of research.

	Experiment No.	Composite-Based Actuator			Experiment No.	Aluminum-Based Actuator		
		y_set_ (mm)	t_e_ (s)			y_set_ (mm)	t_e_ (s)	
Unimorph	37 to 38	0.84	2.5	0.5	39 to 40	0.57	2.5	0.5
	41 to 42	0.64	2.5	0.5	43 to 44	0.45	2.5	0.5
	45 to 46	0.42	2.5	0.5	47 to 48	0.33	2.5	0.5
	49 to 50	0.20	2.5	0.5	51 to 52	0.21	2.5	0.5
Bimorph	53 to 54	1.12	2.5	0.5	55 to 56	0.85	2.5	0.5
	57 to 58	0.87	2.5	0.5	59 to 60	0.69	2.5	0.5
	61 to 62	0.59	2.5	0.5	63 to 64	0.49	2.5	0.5
	65 to 66	0.31	2.5	0.5	67 to 68	0.29	2.5	0.5

**Table 6:** Value of quality index I_q_.

	Experiment No.	Composite-Based		Experiment No.	Aluminum-Based	
		I_q_ (−)			I_q_ (−)	
Unimorph	37 to 38	0.837	0.822	39 to 40	0.942	0.919
	41 to 42	0.852	0.835	43 to 44	0.958	0.928
	45 to 46	0.916	0.881	47 to 48	1.011	0.963
	49 to 50	1.016	0.941	51 to 52	1.033	0.951
Bimorph	53 to 54	0.786	0.777	55 to 56	0.864	0.849
	57 to 58	0.784	0.771	59 to 60	0.878	0.857
	61 to 62	0.793	0.767	63 to 64	0.895	0.870
	65 to 66	0.859	0.816	67 to 68	0.940	0.887

The lower the value of the I_q_ index, the better the control quality.

## Data Availability

Data are contained within the article.
